# Mechanisms and Disease Pathogenesis Underlying Metal-Induced Oxidative Stress

**DOI:** 10.1155/2018/7612172

**Published:** 2018-09-18

**Authors:** Pan Chen, Julia Bornhorst, M. Diana Neely, Daiana S. Avila

**Affiliations:** ^1^Department of Molecular Pharmacology, Albert Einstein College of Medicine, Bronx, NY, USA; ^2^Department of Food Chemistry, Institute of Nutritional Science, University of Potsdam, Nuthetal, Germany; ^3^Department of Pediatrics, Vanderbilt University Medical Center, Nashville, TN, USA; ^4^Graduation Program in Biochemistry, Federal Pampa University, Uruguaiana, RS, Brazil

Metals are involved in almost all aspects of human life today: some are essential for maintenance of cell physiological activities and others are important industrial materials. Over half of the elements (23) with known physiological functions in humans are metals (12), including iron, zinc, copper, and manganese. While the deficiency of some metals may cause pathological conditions (such as iron-deficiency anemia and restless legs syndrome), excess exposure to metals can be toxic and can contribute to human diseases, including cancer, hepatic diseases, and neurodegenerative diseases (such as Alzheimer's, Parkinson's, and Huntington's diseases). Metals such as mercury and lead that are widely used in modern industry do not have any known physiological function in human; however, they are potent neurodevelopmental toxicants. Nowadays, increasing environmental exposures and fortification of food sources with various metallic compounds are causing growing concerns that imbalances in metal content and excess exposure in particular may contribute to the etiology of various human diseases.

Oxidative stress is a fundamental molecular mechanism underlying metal-induced toxicity. Most metals are redox-active, especially transition metals, such as iron, copper, manganese, and zinc. They can undergo redox cycling reactions resulting in the production of reactive oxygen/nitrogen species (RONS). For example, in Fenton's reaction, iron reacts with hydrogen peroxide to produce hydroxyl free radicals, one of the most reactive RONS molecules. Excess intracellular RONS eventually disrupts the intracellular redox state and energy production resulting in oxidative stress, which manifests itself in the modification of cellular biomolecules, such as DNA, lipids and proteins, the dysfunction of mitochondrial respiration, protein folding, DNA repair processes, endoplasmic reticulum (ER) stress, inflammation, autophagy, and/or apoptosis. These cellular phenotypes are frequently observed in the pathogenesis of human diseases.

Despite recent advances in biomedical sciences, the exact mechanisms underlying metal-associated cellular toxicity is in most cases not fully established. The understanding and knowledge of the mechanisms underlying metal-induced toxicities is of paramount importance for the development of antidotes for metal intoxication. This special issue includes papers that explore causative links between metals and human diseases (including cancer and neurological disorders), establish molecular mechanisms underlying metal-induced oxidative stress in the pathogenesis of these diseases, and identify molecular compounds with protective effects against these metals.

Neurodevelopmental- and neurodegenerative disease-associated metal toxicities are of great concern for the industrialized modern world, and several different metals including those that play a role in normal cellular physiology have been implicated to have adverse effects on neurodevelopment or to play a role in the pathogenesis of neurodegenerative or both.

Iron is present in several metalloproteins and, at physiological concentrations, plays a role in enzyme catalysis and in cellular functions such as oxygen transport, mitochondrial respiration, DNA, neurotransmitter, and myelin biosynthesis; however, excess iron has been associated with the pathogenesis of several neurodegenerative diseases, including Alzheimer's and Parkinson's diseases. R. M. Uranga and G. A. Salvador have reviewed the toxic effects of iron overload on the brain and the interaction of this metal with the amyloid *β* peptide. The authors conclude that excess iron results in oxidative stress. They postulate that the exploration of downstream targets of the iron-induced ROS and their role in CNS pathogenesis will allow us to shed light on mechanisms underlying neurodegeneration and the development of therapeutic tools.

Cadmium which has no known physiological function has been described as a serious hazard to human health by the International Agency for Research on Cancer. H. R. Marini et al. set out to assess the effects of chronic cadmium exposure on the mouse hippocampus and to explore the potential protective effects of polydeoxyribonucleotide, an adenosine A2A receptor agonist. They report a cadmium-induced decline of spatial memory and learning, a significant decrease of BDNF expression, an increase in mTOR expression, neuronal loss, and brain edema. Coadministration of the adenosine A2A receptor agonist polydeoxyribonucleotide ameliorated all of those cadmium-induced effects. The authors conclude from this and their previous studies and findings reported by other groups that the cadmium-induced expression of mTOR in the CNS observed here is the result of cadmium-increased ROS production and oxidative stress.

Mercury, another metal which has no biological function, is a well-established central nervous system toxicant. W. A. B. Aragão et al. investigated the effects of a chronic relatively low-dose oral mercury chloride (HgCl_2_) exposure on the rat hippocampal campus. They report a 17-fold increase in hippocampal mercury levels in exposed animals over the baseline levels observed in the control group, suggesting that this metal is efficiently transported across the blood-brain barrier. They observed significant impairments in short- and long-term memory including cognitive impairment. They hypothesize that the elevated oxidative stress observed in the hippocampus led to cytotoxicity and apoptosis, affecting both astrocytes and neurons in the hippocampus.

Aside from its well established neurotoxicity, mercury has also been implicated in cancer. In cancer, abnormal cell growth is traditionally viewed to result from various genetic mutations; however, more recent studies support the hypothesis that the epigenetic regulations (such as DNA methylation, histone modification, transcription, and DNA repair) and especially of promoters also play an important role. In their review, R. Zefferino et al. propose a multistep pathway by which mercury promotes cancer development. Based on their study and other groups' studies, they propose that mercury in an initial step causes an imbalance in the cellular redox equilibrium by interfering with the activity of selenocysteine antioxidant enzymes and other sulfhydryl-containing proteins. This mercury-induced prooxidative state in turn results in the inhibition of gap junction-mediated intercellular communication and a proinflammatory cytokine release, both of which isolate cells from their tissue-specific homeostasis promoting excessive proliferation and inhibiting the immune system's defense of such inappropriate proliferation.

Copper-based Casiopeinas are a group of metal-based antineoplastic drugs designed to have lower generalized toxicity as compared to the alternative drugs cisplatin or doxorubicin. However, these drugs have shown significant cardiac toxicities in canine and rodent models. C. Silva-Platas et al. designed their studies to assess potential mechanisms underlying this cardiomyocyte toxicity of this group of drugs. They observed that in isolated hearts perfused with Casiopeinas, contractile function was inhibited and ATP levels were significantly reduced. Heart mitochondria isolated from Casiopeina-treated animals showed a reduction in mitochondrial membrane potential and calcium retention capacity. In cultured cardiomyocytes, Casiopeinas induced proapoptotic caspases and apoptosis. The authors conclude that the mitochondria are the main target of Casiopeina-induced cardiomyocyte toxicity, where these drugs impair mitochondrial respiratory chain function and induce energetic failure, events that typically result in oxidative stress.

Manganese (Mn) is an essential trace element and acts as a cofactor in enzymes, such as Mn superoxide dismutase thus playing a role in the protection against oxidative stress. However, excessive exposure to this metal is associated with cytotoxicity typically resulting from Mn-induced oxidative stress. L. Li and X. Yang have reviewed current studies addressing the connection between Mn and metabolic disorders that have become a significant health concern in the last decade. These disorders include type 2 diabetes mellitus (T2DM), obesity, insulin resistance, atherosclerosis, nonalcoholic fatty liver disease (NAFLD), and hepatic steatosis which share a common pathology involving the generation of excessive levels of reactive oxygen species (ROS), elevated oxidative stress, and inflammation.

Lead is a ubiquitous pollutant and commonly used in industries; therefore, contamination is common. Even low levels of lead can lead to significant physiological changes. Occupational exposure to this metal has thus to be constantly monitored to ensure a safe working environment for the workers. The study of M. Dobrakowski et al. assessed a cohort of 36 males exposed to lead for 36–44 days. As expected, blood lead levels were increased after their exposure; in addition, small changes of calcium, magnesium, zinc, and copper levels were also observed. The authors further report no change in the activities of catalase and superoxide dismutase and no elevation of malondialdehyde levels, a marker of oxidative stress; however, there was a 46% increase in serum lipid hydroperoxides, another manifestation of oxidative stress targeting lipids.

Metal poisoning is an increasing concern in our modern industrialized world. While environmental regulations need to be regularly updated with additional studies concerning the toxicities of metals, intoxication of humans will remain a serious health hazard at least for the nearer future. Antidotes for metal intoxication are rare, and there is an urgent need to develop therapeutic drugs to counteract metal poisoning. The elucidation of the mechanism underlying metal toxicities will be of a great value to the development of effective metal antidotes. Every study in this issue concludes that oxidative stress is indeed an important metal-induced effect and is the cause that ultimately leads to dysfunction of cellular mechanisms and/or cell death in the model system examined. The mechanism by which a metal induces oxidative stress and the downstream targets of the oxidative stress should be evaluated using a combination of oxidative stress measures so that an “oxidative stress signature” phenotype can be established. It is the hope that such mechanistic analysis and knowledge will be able to support the successful development of therapeutics for the treatment of metal poisonings.

